# Erythropoietin Blockade Inhibits the Induction of Tumor Angiogenesis and Progression

**DOI:** 10.1371/journal.pone.0000549

**Published:** 2007-06-20

**Authors:** Matthew E. Hardee, Yiting Cao, Ping Fu, Xiaohong Jiang, Yulin Zhao, Zahid N. Rabbani, Zeljko Vujaskovic, Mark W. Dewhirst, Murat O. Arcasoy

**Affiliations:** 1 Department of Pathology, Duke University Medical Center, Durham, North Carolina, United States of America; 2 Department of Radiation Oncology, Duke University Medical Center, Durham, North Carolina, United States of America; 3 Department of Medicine, Duke University Medical Center, Durham, North Carolina, United States of America; Ordway Research Institute, Inc., United States of America

## Abstract

**Background:**

The induction of tumor angiogenesis, a pathologic process critical for tumor progression, is mediated by multiple regulatory factors released by tumor and host cells. We investigated the role of the hematopoietic cytokine erythropoietin as an angiogenic factor that modulates tumor progression.

**Methodology/Principal Findings:**

Fluorescently-labeled rodent mammary carcinoma cells were injected into dorsal skin-fold window chambers in mice, an angiogenesis model that allows direct, non-invasive, serial visualization and real-time assessment of tumor cells and neovascularization simultaneously using intravital microscopy and computerized image analysis during the initial stages of tumorigenesis. Erythropoietin or its antagonist proteins were co-injected with tumor cells into window chambers. In vivo growth of cells engineered to stably express a constitutively active erythropoietin receptor EPOR-R129C or the erythropoietin antagonist R103A-EPO were analyzed in window chambers and in the mammary fat pads of athymic nude mice. Co-injection of erythropoietin with tumor cells or expression of EPOR-R129C in tumor cells significantly stimulated tumor neovascularization and growth in window chambers. Co-injection of erythropoietin antagonist proteins (soluble EPOR or anti-EPO antibody) with tumor cells or stable expression of antagonist R103A-EPO protein secreted from tumor cells inhibited angiogenesis and impaired tumor growth. In orthotopic tumor xenograft studies, EPOR-R129C expression significantly promoted tumor growth associated with increased expression of Ki67 proliferation antigen, enhanced microvessel density, decreased tumor hypoxia, and increased phosphorylation of extracellular-regulated kinases ERK1/2. R103A-EPO antagonist expression in mammary carcinoma cells was associated with near-complete disruption of primary tumor formation in the mammary fat pad.

**Conclusions/Significance:**

These data indicate that erythropoietin is an important angiogenic factor that regulates the induction of tumor cell-induced neovascularization and growth during the initial stages of tumorigenesis. The suppression of tumor angiogenesis and progression by erythropoietin blockade suggests that erythropoietin may constitute a potential target for the therapeutic modulation of angiogenesis in cancer.

## Introduction

Cancer progression is influenced by multiple factors including the induction of tumor angiogenesis. Understanding tumor vascularization and growth at its early stages can provide new insights into mechanisms relevant to progression and metastasis, and facilitate the development of novel anti-angiogenic therapies. We have been interested in events that follow immediately after tumor cells are triggered to initiate angiogenesis. Our previous studies provided evidence that angiogenesis induced by tumor cells after implantation in the host begins at a very early stage when the tumor mass contains only 100 to 300 cells [1]–[5]. The induction of tumor angiogenesis is mediated by many regulatory molecules released by tumor and/or host cells and that constitute potential targets of anti-angiogenic therapy. Vascular endothelial growth factor (VEGF), an important regulator of both physiologic and pathologic angiogenesis, has been successfully targeted in pre-clinical tumor models as well as in clinical trials involving cancer patients. However, the benefits of anti-angiogenic therapy can be limited by redundant mechanisms of angiogenesis control, a problem that may potentially be overcome by targeting multiple angiogenic pathways or the use of broad spectrum angiogenic inhibitors [6]. The characterization of novel angiogenic factors and potential targets involved in the induction of tumor vascularization could contribute to the development of more efficacious anti-angiogenic therapeutic approaches.

Erythropoietin (EPO) is the hematopoietic cytokine that regulates the formation of red blood cells by binding to the erythropoietin receptor (EPOR), a member of the cytokine receptor family that is expressed not only in erythroid cells, but also in many non-hematopoietic cell types including vascular endothelial cells and cancer cells [7]. The findings of recent clinical trials reporting that recombinant erythropoietin (rEPO) therapy in some cancer patients may negatively impact recurrence-free survival have raised concerns regarding potential adverse direct effects of erythropoietin in tumors, such as stimulation of the proliferation of cancer cells and/or tumor angiogenesis [8]–[11]. Several preclinical studies have reported direct effects of rEPO on cancer cells- such as activation of intracellular signal transduction or stimulation of proliferation or migration- whereas other studies have found no significant effects of EPO-EPOR on cancer cell proliferation [7], [12]–[15]. In vascular endothelial cells, EPOR expression has been associated with the ability of EPO to stimulate intracellular signaling and to promote angiogenic responses in various experimental models [16]–[18]. EPO has been implicated in the physiologic angiogenesis that occurs in the developing mouse embryo [19], the female genital tract [20], and during wound healing [21]. A more recent study reported that EPO is involved in the pathologic angiogenesis of proliferative diabetic retinopathy [22]. Although a role for EPO in tumor angiogenesis has been suggested [23]–[26], its potential as a target and direct modulator of pathologic tumor neovascularization is not established.

In the present study, we investigated the role of EPO in tumor angiogenesis and progression. As an angiogenesis model, we used fluorescently-labeled mammary carcinoma cells implanted in dorsal skin-fold window chambers, a model that allows non-invasive, direct, and serial visualization and assessment of both tumor cells and pathologic neovascularization simultaneously. This model has been used to evaluate angiogenic factors such as VEGF, angiopoietin-2, basic fibroblast growth factor and candidate angiogenesis inhibitors that target tumor vasculature [1], [2], [5], [27]. The in vivo growth of mammary carcinoma cells was also assessed in mammary fat pad xenografts in athymic nude mice. We find that local rEPO treatment in window chambers or stable expression of a constitutively active EPOR mutant (EPOR-R129C) in tumor cells promotes the induction of tumor angiogenesis and stimulates growth. Conversely, targeting endogenous EPO using recombinant soluble EPOR (sEPOR) or a neutralizing anti-EPO monoclonal antibody co-injected into window chambers with mammary carcinoma cells inhibits the initiation of tumor angiogenesis and delays growth during the initial stages of tumorigenesis. In orthotopic tumor xenograft studies, EPOR-R129C expression significantly promotes tumor growth associated with increased expression of Ki67 proliferation antigen, enhanced microvessel density, decreased tumor hypoxia, and increased phosphorylation of the p44/42 extracellular-regulated kinases ERK1/2. Stable expression in tumor cells of a secreted EPO antagonist protein (R103A-EPO) exerts a remarkable anti-angiogenic effect and impairs primary tumor growth both in window chambers and in the mammary fat pad. These data provide evidence that EPO is an important angiogenic factor that modulates the induction of tumor cell-induced angiogenesis and progression during the initial stages of tumor formation. Suppression of tumor angiogenesis and growth by EPO blockade suggests that EPO may constitute a potential target for the therapeutic modulation of angiogenesis in cancer that warrants further investigation.

## Results

### Erythropoietin treatment in window chambers promotes tumor angiogenesis and growth

**Figure 1:**
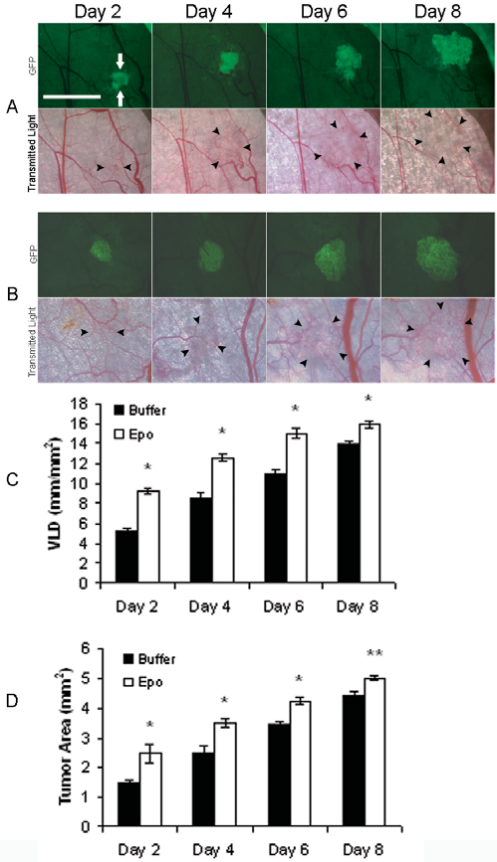
Stimulation of tumor angiogenesis and growth in response to rEPO treatment. Representative images of dorsal skin-fold window chambers implanted with R3230-GFP cells and local administration of (A) control buffer, or (B) recombinant EPO are shown (total n = 8 animals/group). Fluorescent (FITC) and transmitted light images were acquired serially on postoperative days 2, 4, 6, and 8. Scale bar = 2.5 mm. GFP-positive tumor area (green fluorescence) is indicated by white arrows and tumor-associated vasculature in transmitted light images is outlined by black arrowheads. (C) Quantification of tumor neovascularization as measured by vascular length density (VLD) in window chambers treated with EPO or control buffer revealed increased angiogenesis in EPO-treated chambers compared to controls, *P<0.001. (D) Quantification of tumor growth revealed significantly increased tumor area in EPO-treated chambers compared to controls, *P<0.001; ** P<0.01.

Fluorescently-labeled R3230-GFP mammary carcinoma cells were co-injected with erythropoietin into window chambers. Tumor growth and neovascularization were assessed serially using intravital microscopy. Representative fluorescent and transmitted light images of the window chambers are illustrated (figure 1A, B). Quantification of tumor neovascularization revealed significant stimulation of angiogenesis in erythropoietin-treated window chambers compared to buffer-treated negative controls (figure 1C, table 1). The vascular length density increased by 78% compared to buffer-treated controls as early as day 2 after tumor cell injection (P<0.001). Quantification of two-dimensional tumor growth in window chambers revealed that the proangiogenic effect of erythropoietin was associated with a significant increase in tumor growth by 66% and 42% on days 2 and 4, respectively, compared to controls (P<0.001, figure 1D, table 2). The pro-angiogenic and growth promoting effects diminished with time but remained significant by day 8. There was no significant systemic effect of local erythropoietin administration in the window chambers on the hematocrit levels of the animals (47.6±1.2% in erythropoietin-treated compared to 47.7±3.1% in controls).

**Table 1:** Vascular length density measurements in window chambers (mm/mm^2^).

Time	Recombinant EPO		EPOR-R129C^a^		Soluble EPOR and anti-EPO mab			R103A-EPO^b^	
	**Buffer n = 8**	**rEPO n = 8**	**Vector n = 7**	**R129C n = 7**	**Control n = 7**	**sEPOR n = 7**	**mab n = 7**	**Vector n = 7**	**R103A n = 7**
**Day 2**	5.18±0.2	9.26±0.1	4.74±0.1	8.35±0.2	4.72±0.1	3.57±0.3	3.34±0.2	5.96±0.2	3.75±0.1
**Day 4**	8.54±0.4	12.56±0.2	8.94±0.2	13.28±0.2	8.67±0.9	5.8±0.2	5.65±0.3	11.1±0.3	5.58±0.2
**Day 6**	10.96±0.2	14.99±0.3	12.58±0.2	14.67±0.2	12.34±0.7	7.41±0.3	6.64±0.3	13.62±0.4	6.81±0.2
**Day 8**	13.96±0.2	15.92±0.2	14.92±0.2	16.37±0.2	14.72±0.4	8.67±0.5	7.2±0.5	16.03±0.4	7.98±0.3

a Two independent clones each of vector (pCR3.1) and EPOR-R129C transfected cells were used (3+4 = 7 animals/group).

b Two independent clones each of vector (pCDNA3.1) and R103A-EPO transfected cells were used (3+4 = 7 animals/group).

**Table 2:** Tumor area measurements in window chambers (mm^2^).

Time	Recombinant EPO		EPOR-R129C^a^		Soluble EPOR and anti-EPO mab			R103A-EPO^b^	
	**Buffer n = 8**	**rEPO n = 8**	**Vector n = 7**	**R129C n = 7**	**Control n = 7**	**sEPOR n = 7**	**mab n = 7**	**Vector n = 7**	**R103A n = 7**
**Day 2**	1.47±0.06	2.45±0.21	1.51±0.11	2.14±0.26	2.01±0.07	1.54±0.13	1.84±0.12	1.43±0.14	0.81±0.03
**Day 4**	2.44±0.2	3.48±0.09	2.19±0.15	3.20±0.34	3±0.1	3.06±0.27	2.81±0.22	2.49±0.13	0.79±0.05
**Day 6**	3.42±0.07	4.24±0.08	2.73±0.14	4.47±0.34	4.47±0.26	3.39±0.24	3.45±0.34	3.44±0.3	0.73.±0.07
**Day 8**	4.36.±0.14	5±0.05	3.12±0.13	5.58±0.31	5.59±0.21	3.36±0.29	3.06±0.25	4.35±0.4	0.6±0.07

a Two independent clones each of vector (pCR3.1) and EPOR-R129C transfected cells were used (3+4 = 7 animals/group).

b Two independent clones each of vector (pCDNA3.1) and R103A-EPO-transfected cells were used (3+4 = 7 animals/group).

### Expression of constitutively active EPOR-R129C in mammary carcinoma cells is associated with increased angiogenic response and tumor growth

**Figure 2:**
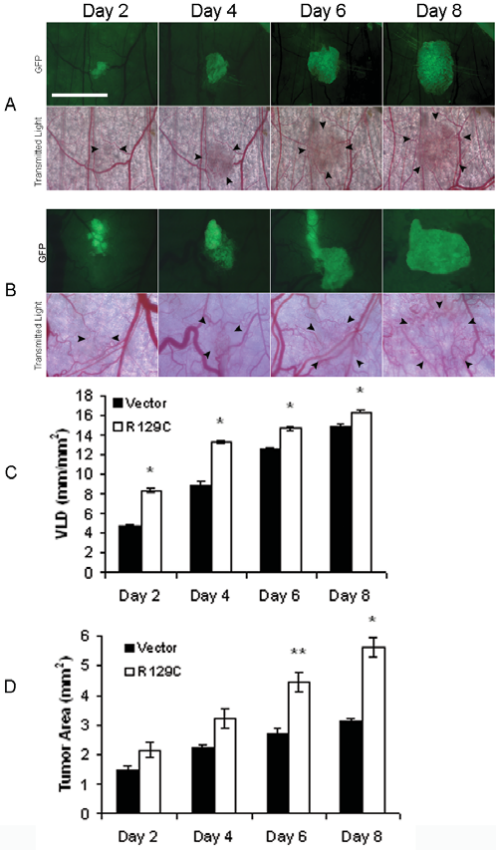
Stimulation of tumor angiogenesis and growth in response to tumor cell EPOR-R129C expression. Representative images of dorsal skin-fold window chambers implanted with R3230-GFP cells transfected with (A) empty pCR3.1 vector control, or (B) EPOR-R129C expression vector are shown. Scale bar = 2.5 mm. Two independent single cell clones each of empty pCR3.1 vector and EPOR-R129C-transfected cells were tested for a total of 7 animals in each group. (C) Quantification of tumor neovascularization in window chambers implanted with EPOR-R129C or empty vector-transfected R3230-GFP cells revealed significantly increased VLD in EPOR-R129C expressing group compared to vector controls, *P<0.001. (D) Quantification of tumor growth in window chambers implanted with EPOR-R129C or vector transfected R3230-GFP cells revealed increased tumor area in EPOR-R129C expressing group compared to vector controls, *P<0.001; **P<0.01 (n = 7 animals/group).

To further investigate the role of erythropoietin and its receptor in tumor angiogenesis and progression, R3230-GFP cells were engineered to express constitutively active EPOR-R129C, a mutant EPOR that confers growth factor-independent proliferation and tumorigenicity when expressed in immortalized hematopoietic cells but does not transform normal fibroblasts [28]–[31]. Single cell clones of R3230-GFP cells expressing EPOR-R129C were isolated (figure S1) and in vitro growth rates and cell cycle profile of the cells were characterized. R3230-GFP cells expressing EPOR-R129C exhibited similar growth characteristics in serum-containing medium compared to vector-transfected negative control cells (figure S1). When implanted into window chambers and monitored serially by fluorescent and transmitted light intravital microscopy, EPOR-R129C expression was associated with a significant enhancement of tumor-cell induced neovascularization and growth in all chambers (figure 2A, B). There was a significant increase in vascular length density by 76% and 48% on days 2 and 4, respectively, compared to controls (P<0.001, figure 2C, table 1). This proangiogenic effect was associated with a significant increase in tumor growth by 63% and 79% at day 6 and day 8, respectively (P<0.001, figure 2D, table 2).

**Figure 3:**
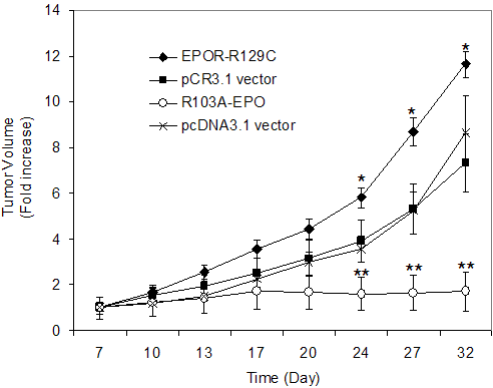
In vivo tumor growth of R3230 mammary carcinoma cells in the mammary fat pad of nude mice. Stably transfected R3230-GFP cells were injected into the mammary fat pad of mice, tumor volumes were measured and expressed as fold-increase of palpable tumor size at day 7. Three independent single cell clones of each cell line were analyzed and empty vector-transfected cells (pCR3.1 and pcDNA3.1) served as negative controls. Expression of EPOR-R129C was associated with a significant increase in tumor growth rate compared to pCR3.1 vector controls (*P<0.001, n = 19 tumors in EPOR-R129C and n = 16 in pCR3.1 group). No significant tumor growth was observed in animals injected with cells expressing R103A-EPO antagonist (n = 14) compared to pcDNA3.1 vector transfected cells (**P<0.001, n = 15 tumors in pcDNA3.1 group).

To further characterize the in vivo growth of R3230-GFP cells expressing EPOR-R129C, orthotopic tumor xenograft experiments were performed by injecting the mammary carcinoma cells into the mammary fat pad of athymic nude mice. Three independent single cell clones of each stably transfected cell line were analyzed and empty pCR3.1 vector-transfected cells served as negative controls. Tumor growth rate was significantly increased in animals injected with cells expressing EPOR-R129C compared to the growth rate in animals injected with negative control cells (P<0.001, figure 3, table 3).

**Table 3:** Tumor volume measurements in mammary fat pad (mm^3^).

Day	pCR3.1 vector **(n = 16)**	EPOR-R129C **(n = 19)**	pcDNA3.1 vector **(n = 15)**	R103A-EPO **(n = 14)**
7	33.7±9.1	43.6±6.3	18.9±4.7	14.7±7
10	52.5 ±13.8	73.3±9.6	21.8±5.2	18.2±8.7
13	65.1±16.7	111.5±12.5	28.8±6.4	21.2±10.2
17	84.7±21.3	155.1±16.8*	43.1±7.2	25.6±11.9
20	106.3±25.9	192.8±19.9*	56.3±8.4	24.6±11**
24	131.2±30.9	253.1±18.7*	67.6±10.8	23.6±10.7**
27	179±36.5	378.7±26.9*	99.3±14.5	24.1±11.5**
32	247±42.8	507.3±24.9*	163.1±30.5	25.2±12.6**

* p<0.05 compared to pCR3.1 vector control.

** p<0.05 compared to pcDNA3.1 vector control.

### Erythropoietin blockade using recombinant soluble EPOR and anti-EPO monoclonal antibody inhibits tumor angiogenesis and delays growth in window chambers

**Figure 4:**
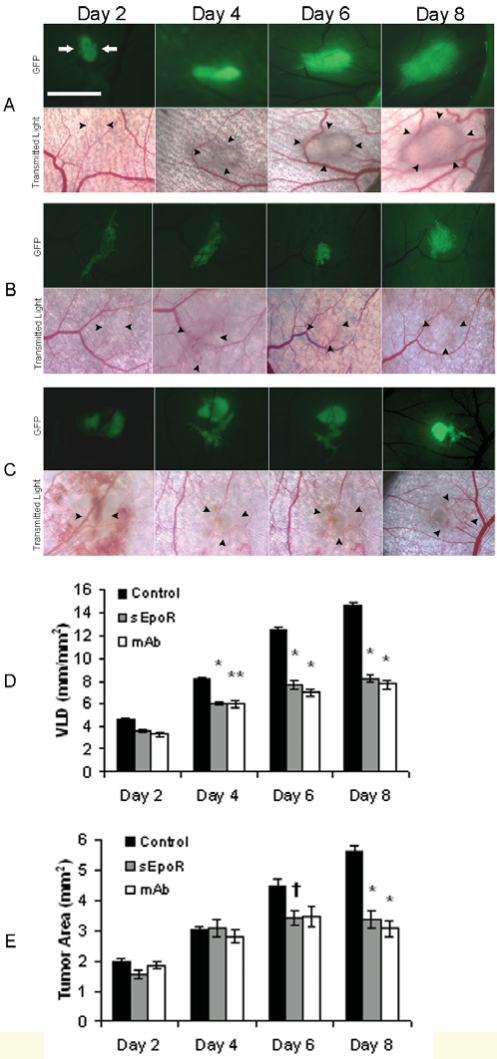
Erythropoietin blockade using soluble EPOR or anti-EPO mab suppress tumor angiogenesis and delay growth. (A–C) Representative images of window chambers implanted with R3230-GFP cells co-injected with (A) control buffer or protein, (B) sEPOR, or (C) anti-EPO mab. Fluorescent and transmitted light images were acquired serially on postoperative days 2, 4, 6, and 8. Scale bar = 2.5 mm. (D) Quantification of tumor neovascularization revealed significantly decreased angiogenesis in sEPOR and mab-treated chambers compared to controls. Control buffer was PBS (n = 3) and control protein was mouse IgG1 (n = 4), *P<0.001; **P<0.01, (n = 7 animals/group). (E) Quantification of tumor growth revealed significantly decreased tumor area in sEPOR and mab-treated chambers compared to controls, *P<0.001; ^†^P<0.05, (n = 7 animals/group).

We investigated whether protein inhibitors targeting EPO function could suppress tumor-cell induced neovascularization and delay growth. The antagonists recombinant sEPOR and neutralizing monoclonal anti-EPO antibody (mab) inhibit EPO-dependent proliferation of hematopoietic 32D cells that express the EPOR (figure S2). We determined the in vivo effect of the antagonist proteins on angiogenesis and tumor growth in window chambers. Local, one-time treatment with the antagonist proteins, co-injected with R3230-GFP tumor cells into window chambers, resulted in significant inhibition of neovascularization and tumor growth delay (figure 4A, B, C). The negative controls were treated with mouse IgG1 (4 windows) or phosphate-buffered saline (3 windows). Compared to controls, significant inhibition of tumor angiogenesis was observed leading to decreased vascular length density by 44% (sEPOR) and 47% (mab) at day 8 (P<0.001, figure 4D, table 1). This anti-angiogenic effect was associated with significant tumor growth delay with decreased two-dimensional tumor size by 37% (sEPOR) and 39% (mab) at day 8 (P<0.001, figure 4E, table 2).

### R103A-EPO antagonist secretion from mammary carcinoma cells blocks induction of tumor neovascularization and inhibits tumorigenesis

**Figure 5:**
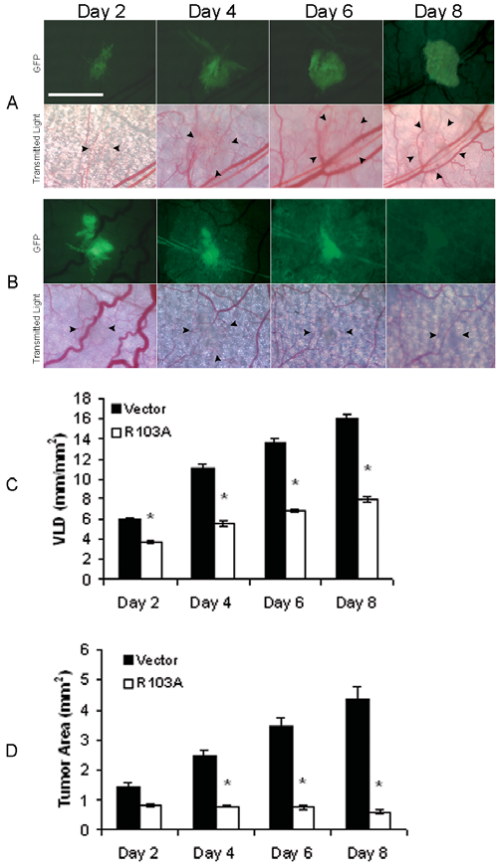
Expression of R103A-EPO antagonist inhibits induction of tumor angiogenesis and disrupts primary tumor growth. Representative images of window chambers implanted with R3230-GFP cells transfected with (A) empty pcDNA3.1 vector, or (B) R103A-EPO antagonist expression vector. Note the constriction and near-complete disappearance of blood vessels in the areas surrounding the tumor which was observed in all window chambers injected with cells expressing R103A-EPO. Two independent single cell clones each of empty pcDNA3.1 vector or R103A-EPO-transfected cells were analyzed for a total of 7 animals in each group. (C) Quantification of tumor neovascularization revealed decreased angiogenesis in window chambers implanted with R103A-EPO secreting cells compared to vector controls, *P<0.001. (D) Quantification of tumor growth in window chambers implanted with R103A-EPO or vector transfected R3230-GFP cells. Compared to vector controls, decreased tumor area was observed in R103A-EPO expressing group with near-complete disappearance of tumor cells at day 8 in all chambers, *P<0.001 (n = 7).

To target erythropoietin using a different strategy, R3230-GFP cells were engineered to constitutively express and secrete mutant erythropoietin protein R103A-EPO (figure S3). R103A-EPO is an antagonist capable of inhibiting erythropoietin-dependent proliferation of hematopoietic cells acting as a competitive inhibitor of erythropoietin [32]. The in vitro growth rates and cell cycle profile of R3230-GFP cells expressing R103A-EPO antagonist were similar to vector-transfected negative control cells (figure S1). We previously showed that rEPO treatment induces the phosphorylation of the extracellular-regulated kinases ERK1/2 in R3230 cells [33]. Expression of the antagonist R103A-EPO had no significant effect on the ability of rEPO to induce the increased phosphorylation of ERK1/2 in mammary carcinoma cells (figure S3). To determine the in vivo effects of secreted R103A-EPO antagonist, tumor cells expressing R103A-EPO were implanted into window chambers and angiogenesis induction and tumor growth were monitored. Compared to empty vector-transfected cells, there was striking inhibition of tumor-cell induced angiogenesis and disruption of tumor formation associated with R103A-EPO antagonist expression (figure 5A, B). Vascular length density was significantly inhibited by 50% as early as day 4, with the significant anti-angiogenic effect maintained throughout 8 days of observation and measurements (P<0.001, figure 5C, table 1). There was severe constriction and tapering of the blood vessels in areas surrounding the tumor. Furthermore, this striking anti-angiogenic effect was associated with disordered tumor growth and significant growth inhibition by 85% on day 8 when near-complete disappearance of the implanted tumor cells was observed in all chambers (P<0.001, figure 5D, table 2). There was no systemic effect of tumor cell R103A-EPO antagonist expression on hematocrit levels of the animals (48.8±1.3% compared to 47.3±0.4 % in controls).

The in vivo growth of R3230-GFP cells expressing R103A-EPO antagonist was further characterized in orthotopic tumor xenograft experiments by inoculating the tumor cells in the mammary fat pad of athymic nude mice. Three independent single cell clones of the stably transfected cell lines were analyzed and empty pCDNA3.1 vector-transfected cells served as negative controls. As illustrated in Figure 3, primary tumor growth was severely impaired in the animals injected with cells expressing R103A-EPO, compared to the expected tumor growth in animals injected with negative control cells (P<0.001, table 3).

### Enhanced EPO-induced phosphorylation of ERK1/2 and c-Jun-NH2-kinase (JNK) in mammary carcinoma cells expressing EPOR-R129C

**Figure 6:**
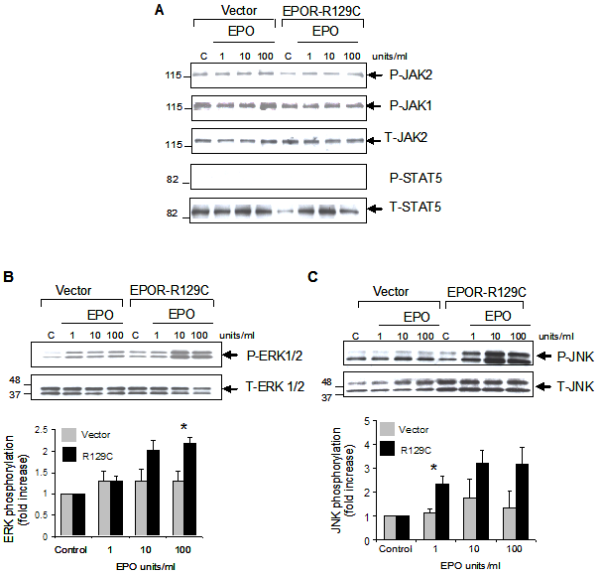
Increased EPO-induced phosphorylation of ERK1/2 and JNK in mammary carcinoma cells expressing EPOR-R129C. R3230-GFP cells transfected with empty pCR3.1 vector or EPOR-R129C were either left untreated as controls (C) or incubated with the indicated concentration of rEPO for 10 minutes. Whole cell lysates were analyzed by Western blotting using antibodies against phosphorylated or total proteins to detect (A) JAK2, JAK1 and STAT-5, (B) ERK1/2, and (C) JNK. Representative blots are shown. The phosphorylated (P) and total (T) proteins are indicated by the arrows. Comparable sample loading and protein integrity were confirmed by stripping the blots and hybridizing to respective antibodies to detect total protein amounts in the samples. The relative positions of the molecular weight markers are indicated in each blot. Quantitative representation of phosphorylated proteins using densitometry normalized to total protein levels are shown below the blots for ERK1/2 and JNK (n =  3 independent experiments using 3 different single cell clones, (*P<0.05).

We evaluated the activation of EPO-dependent signal transduction in R3230 mammary carcinoma cells engineered to express the constitutively active EPOR-R129C mutant. Hematopoietic cells expressing EPOR-R129C have been reported to exhibit constitutive as well as EPO-inducible phosphorylation and activation of the cytoplasmic tyrosine kinase JAK2, its substrate STAT5 and extracellular-regulated kinases ERK1/2 [34]–[37]. However, in mammary carcinoma cells, we did not observe any significant changes in the tyrosine phosphorylation of JAK2 in cells expressing EPOR-R129C. Moreover, rEPO treatment failed to induce the tyrosine phosphorylation of JAK2 or STAT5 in mammary carcinoma cells (figure 6A), even following longer time-course stimulation of the cells with rEPO (data not shown). Failure of rEPO to induce the tyrosine phosphorylation of JAK2 or STAT5 was also observed in human breast cancer cell lines MCF-7 and MDA-MB-231 (data not shown). In a non-hematopoietic myoblast-like cell line, EPO has been previously reported to induce the tyrosine phosphorylation of JAK1 [38], however, rEPO did not induce the tyrosine phosphorylation of JAK1 in mammary carcinoma cells (figure 6A). We found that rEPO treatment induced the increased phosphorylation of ERK1/2 and c-Jun-NH2-kinase (SAPK/JNK) which were significantly enhanced in cells expressing EPOR-R129C (figure 6B, C). Despite increased ERK1/2 and JNK phosphorylation, rEPO treatment of the cells did not result in consistent stimulation of cell proliferation in vitro (data not shown).

### Tumor cell EPOR-R129C expression is associated with increased in vivo ERK1/2 phosphorylation, Ki67 proliferation antigen expression and microvessel density

**Figure 7:**
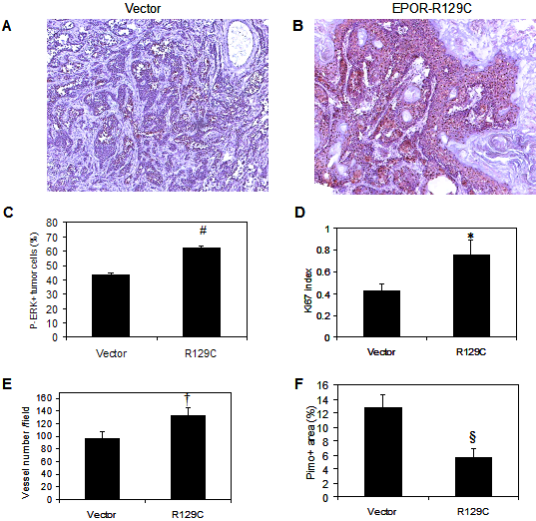
Effect of tumor EPOR-R129C expression on in vivo ERK1/2 phosphorylation, Ki67 proliferation antigen expression, microvessel density and hypoxia. Sections of pCR3.1 vector control and EPOR-R129C mammary fat pad tumors were analyzed by immunocytochemistry as described in Materials and Methods. (A–C) Phospho-ERK, (D) Ki67, (E) CD31, and (F) Pimonidazole hypoxia marker. #P<0.0001;*P = 0.0006; †P = 0.0008; § P = 0.026.

We then investigated whether EPOR-R129C expression may be associated with increased in vivo phosphorylation of ERK1/2 in mammary fat pad tumors by immunocytochemical staining. Representative photomicrographs of empty vector and EPOR-R129C tumors are shown (figure 7A, B). In EPOR-R129C tumors, there was a significant increase in phospho-ERK1/2 positive tumor cells compared to vector controls (P<0.0001, figure 7C). The presence of increased phospho-ERK in EPOR-R129C tumors was associated with increased Ki67 proliferation antigen expression (P = 0.0006, figure 7D). Assessment of tumor angiogenesis revealed a significant increase in microvessel density in EPOR-R129C tumors compared to vector controls (P = 0.0008, figure 7E) and the increased microvessel density in EPOR-R129C tumors was associated with significant decrease of tumor hypoxia as determined by immunostaining for pimonidazole adduct formation (P = 0.026, figure 7F).

## Discussion

The present studies show that erythropoietin is an important angiogenic factor that regulates the induction of tumor neovascularization. A key aspect of our studies is the direct, serial and simultaneous visualization and assessment of both implanted tumor cells and host blood vessels to show that endogenous erythropoietin is a critical factor involved in the attraction of new capillaries to vascularize the expanding tumor mass. Using three different inhibition strategies targeting endogenous EPO-EPOR - including the administration of sEPOR and anti-EPO mab proteins as well as tumor cell R103A-EPO antagonist expression- the data demonstrates that erythropoietin blockade can effectively inhibit the host angiogenic response and thereby severely impair primary tumor growth. The magnitude of the anti-angiogenic and anti-tumor effects of erythropoietin blockade using R103A-EPO expression is comparable to that achieved by targeting VEGF in window chambers in our previous experiments [2], [5]. Thus, our current findings suggest that the EPO-EPOR system may constitute a potential target for the therapeutic modulation of angiogenesis in cancer that warrants further investigation.

Here, we find that local rEPO administration induces a significant proangiogenic effect and a transient stimulation of tumor growth in window chambers. In previous studies, systemically administered rEPO over a 3 to 4 week period did not enhance the growth of various types of tumor xenografts, including R3230 tumors, despite the ability of erythropoietin to activate intracellular signaling in the cells [33], [39], [40]. It is possible that the pro-angiogenic properties and modest growth-promoting effects of EPO require sustained and high local concentrations in the tumor microenvironment that cannot be achieved by systemic, intermittent rEPO administration. To further investigate the role of tumor cell EPOR expression, we over-expressed the constitutively active EPOR-R129C in R3230 cells and found significantly increased tumor growth rate in both window chambers and orthotopic xenografts, demonstrating that EPOR over-expression and signaling in mammary carcinoma cells is capable of promoting growth. The over-expression of EPOR-R129C in cancer cells was specifically associated with the increased phosphorylation of ERK1/2 both in vitro and in vivo. In contrast to the well-established signaling pathway activation mediated by EPO-EPOR in hematopoietic cells, EPO treatment of mammary carcinoma cells did not induce the phosphorylation of the JAK2/STAT5 axis- a critical pathway for physiologic EPOR function in hematopoietic cells [41]. These findings suggest the presence of significant differences between EPOR-mediated signaling in hematopoietic cells compared to tumor cells and that the level of EPOR expression in tumor cells may modulate cellular responses to exogenous EPO. Thus, the development of novel reagents and techniques to accurately quantify EPOR expression in primary human tumors is required as a tool to further characterize EPO biology in cancer.

An important finding of our studies is that EPO blockade in window chambers disrupted tumor neovascularization and growth, particularly in the window chambers implanted with R3230-GFP cells secreting the R103A-EPO antagonist which was associated with virtual disappearance of tumor-associated blood vessels, and eventually, the implanted tumor cells. Although the window chamber angiogenesis model provides the advantage of direct visualization of both tumor cells and host vasculature simultaneously, it is limited by the requirement of growing tumors in an ectopic site and the two-dimensional assessment of tumor growth. Therefore, we examined the growth of mammary carcinoma cells injected into the mammary fat pad and found an even more dramatic effect of erythropoietin blockade in our tumor xenograft studies, where R103A-EPO antagonist expression in cancer cells almost completely abrogated primary *in vivo* tumor formation. Suppression of *in vivo* tumor growth in window chambers and in the orthotopic site occurred despite similar *in vitro* proliferation rate of R103A-EPO expressing cells compared to controls (figure S1). The remarkable anti-angiogenic effect associated with R103A-EPO-secreting tumor cells in window chambers and the disruption of tumor growth in the mammary fat pad suggest the presence of a paracrine inhibitory effect of R103A-EPO antagonist on the host neovascularization response. Taken together, these findings are consistent with the dependence of individual tumor cells on early angiogenic activity for both survival and proliferation *in vivo* and provide evidence for the critical role for endogenous EPO-EPOR in this process.

Our findings suggest that further investigation is warranted to employ erythropoietin blockade for the therapeutic modulation of tumor angiogenesis. The therapeutic efficacy of different inhibitors of angiogenesis may vary depending on the stage of carcinogenesis during which the inhibitors are employed [42]. Using the dorsal skin-fold window chamber angiogenesis model, we focused on erythropoietin blockade during the initial stages of tumorigenesis by employing local antagonists targeting erythropoietin function. Whether systemic treatment with agents targeting erythropoietin, such as purified R103A-EPO antagonist protein, can produce regression of high tumor burdens and/or prevent metastasis remains to be determined. The systemic administration of putative anti-angiogenic agents targeting erythropoietin and its receptor may be limited by the development of anemia due to the suppression of erythropoiesis. Although local erythropoietin blockade in tumors may represent one approach to overcome this problem, selective inhibition of EPOR function to specifically target cancer cells may constitute an alternative future strategy. In a series of recent studies, erythropoietin has emerged as a major tissue-protective cytokine against various types of injury in diverse non-hematopoietic tissues [43]–[45]. The characterization of EPO variants that mediate the non-hematopoietic biologic effects of EPO without inducing signaling in hematopoietic cells [46] suggests that strategies to develop EPO-EPOR antagonists to selectively target non-hematopoietic cells- possibly including cancer cells and tumor vasculature- without causing anemia might be feasible, particularly when the exact structure of the cell surface receptor that mediates erythropoietin signaling in non-hematopoietic cells is ascertained [47].

## Materials and Methods

### Reagents

Recombinant erythropoietin (Procrit) was purchased from the outpatient pharmacy at Duke University Medical Center. Recombinant soluble EPOR (307-ER-050), neutralizing monoclonal antibody against EPO (mab287) and murine IgG1 as negative control antibody (mab002) were from R&D Systems (Minneapolis, MN) and were reconstituted in phosphate-buffered saline (PBS). Polyclonal anti-EPO (H-162), anti-EPOR (M-20), anti-JAK2 (C-20), anti-phospho-JAK2 (Tyr1007/1008), anti-STAT5 (N-20) antibodies were from Santa Cruz Biotechnology (Santa Cruz, CA). Phospho-ERK1/2 (Thr202/Tyr204), total ERK1/2, phospho-JNK (Thr183/Tyr185), total JNK, phospho-STAT5 (Tyr694), phospho-JAK1 (Tyr1022/1023) antibodies were from Cell Signaling Technologies (Beverly, MA). The mutant erythropoietin R103A-EPO antagonist cDNA [48], [49] and mammalian expression vector (pcDNA3.1) were provided by Dr. H. Franklin Bunn and Dr. Kevin W. Harris. The constitutively active mutant EPOR-R129C cDNA [28]–[31], [34]–[36] was provided by Dr. Harvey F. Lodish and cloned into pCR3.1 vector (Invitrogen). All plasmid sequences were confirmed by sequencing at the Duke University Medical Center DNA sequencing facility using an ABI PRISM^(TM)^ 377 DNA sequencer and Dye Terminator Cycle Sequencing system (Perkin-Elmer).

### Cell culture, transfections and in vitro proliferation assays

R3230 rat mammary carcinoma cells that constitutively express GFP (R3230-GFP) were maintained in RPMI medium supplemented with 10% fetal bovine serum (Hyclone). R3230-GFP cells sensitive to G418 were transfected with expression plasmids using Lipofectamine reagent (Invitrogen, Carlsbad, CA) and 48 hours later, cells were cultured in fresh medium containing 400 µg/ml G418 (Invitrogen) to select resistant single cell clones isolated in cloning chambers. Negative controls included mock transfected cells that underwent 100% cell death in G418. In addition, cells were transfected with empty expression vectors to isolate single cell clones of G418-resistant, negative control cell lines. In vitro growth curves of transfected cell lines were generated by daily counts of cells plated in triplicates (5×10^4^ cells/well) in serum-containing medium in 6-well plates. Culture media was changed every day. Cells were trypsinized and counted daily using a Coulter Counter. Cell cycle analysis was performed during exponential growth in serum-containing medium by propidium iodide staining and FACS analysis. Cells were trypsinized, fixed with ice-cold ethanol, and stained with propidium iodide (Sigma) in the presence of RNAse (Qiagen). Cells were then subjected to flow cytometric analysis to determine the fractions of cells in subdiploid, G1, S, and G2/M phase populations. Hematopoietic 32D cells that were transfected to express EPOR were described previously [50]. To measure inhibition of EPO-dependent proliferation by protein antagonists, 32D cells were cultured in phenol-free medium in 96-well plates (5×10^4^ cells/well) for 3 days in the presence of 0.3 units/ml of rEPO and increasing concentrations of either sEPOR or neutralizing anti-EPO mab-287. Cells were incubated with MTT reagent (dimethylthiazol-2-yl-2,5-diphenyltetrazolium) for 3 hours at 37°C, solubilized in isopropanol:hydrochloric acid (24∶1) solution and the optical density (562–650 nm) was determined using a microtitre plate reader.

### Protein analyses

To prepare whole cell lysates, cells were washed with ice-cold phosphate-buffered saline and lysed in a buffer containing 1% Triton-X-100, 10% glycerol, 150 mM NaCl, 20 mM Tris-HCl (pH 7.4), 5 mM EDTA, supplemented freshly with leupeptin 10 µg/ml, aprotinin 10 µg/ml, pepstatin 1 µg/ml, 1 mM PMSF, 1 mM Na_3_VO_4_ and 10 mM NaF. In some experiments, cells were starved free of serum for one hour and then incubated with rEPO prior to lysis. The cell lysates were cleared by centrifugation at 16,000×*g* for 15 minutes and protein concentration in the supernatants was determined by the Bio-Rad protein assay. The proteins were denatured at 95°C for 4 minutes in Laemmli sample buffer and separated by SDS-polyacrylamide gel electrophoresis (PAGE) using 4–12% Tris-glycine gels (Novex). The separated proteins were subsequently transferred to Immobilon PVDF membranes (Millipore), blocked with 5% non-fat milk in TBST (20 mM Tris-HCl, 137 mM NaCl, 0.1% Tween-20, pH 7.5) for 1 hour and incubated with primary antibodies at 4°C overnight followed by horseradish peroxidase-conjugated secondary antibodies. Proteins were detected using enhanced chemiluminescence and bands were visualized by autoradiography. As positive control for EPOR expression, protein extracts of spleen tissue previously harvested from phenylhydrazine-treated anemic mice was used [51]. To detect secreted R103A-EPO in cell culture supernatants, single cell clones of stably transfected R3230-GFP cells were cultured in fresh medium for 48 hours and an aliquot of the culture medium was analyzed directly by SDS-PAGE and immunoblotting.

### Animals

Female athymic nude mice (BALB/c nu/nu) 8–10 week old were obtained from the National Cancer Institute and housed in a temperature-controlled animal-care barrier facility with a 12-h light-dark cycle. All procedures were approved by and in accordance with the guidelines of Duke Institutional Animal Care and Use Committee and the National Institutes of Health Guide for the Care and Use of Laboratory Animals (NIH publication No. 85-13, revised 1996). Animals were treated in a humane fashion and all efforts were made to minimize animal suffering and the number of animals used.

### Dorsal skin-fold window chambers and tumor cell injection

Nude mice were anesthetized with a mixture of ketamine (100 mg/kg, i.p.) and xylazine (10 mg/kg i.p.), and window chamber surgeries were performed under sterile conditions [2]. A 5 mm diameter flap of skin was dissected away from opposing surfaces of the dorsal skin fold, leaving a fascial plane with associated vasculature. The hole was held vertically away from the body with a titanium saddle that was sutured to both sides of the flap. Tumor cells (5×10^3^ cells/10 µl) were injected into the remaining fascial layer using a Hamilton syringe and glass windows were attached to the center of the saddle to cover the surgical site. Treatments were co-injected with the tumor cells during the surgeries and included recombinant sEPOR (8 µg/injection) or anti-EPO mab (40 µg/injection) at doses that were about eight-fold higher than that required to maximally inhibit EPO-dependent in vitro proliferation of hematopoietic cells (figure S2). As negative control for these treatments, mouse IgG_1_ protein (40 µg/injection) was injected in 4 window chambers and phosphate buffered saline was injected in 3 windows. In previous studies, injection of bovine serum albumin had no effect on angiogenesis and tumor growth [2]. Erythropoietin (280 units/treatment) in a volume of 7 µl was combined with hydron [poly(2-hydroxyethyl methacrylate); 12% in ethanol; Sigma], and dried in a desiccator at 4°C to form a transparent pellet, which was implanted adjacent to the tumor cell injection site in the window chamber. The concentrated form of pharmaceutical grade rEPO (Procrit, 40,000 units/ml) permitted the maximum administration of 280 units into each window chamber. The formulation buffer in pellet form was used as a negative control for erythropoietin treatments and consisted of 100 mM NaCl, 20 mM sodium citrate, 0.3 mM citric acid [pH 6.9] and 2.5 mg/ml human albumin (Sigma).

### Intravital microscopy, tumor growth and angiogenesis assessments

Window chambers were imaged using a Carl Zeiss MPS intravital microscope (Carl Zeiss, Hanover, MD) equipped with a color video camera (Carl Zeiss ZVS-3C75DE) connected to a PC computer with a frame grabber and Scion Image software (Scion Corporation, Frederick, MD). Fluorescence epi-illumination was provided with a 100 W mercury-arc lamp (AttoArc HBO, Carl Zeiss, Inc.) and FITC filter (excitation 450–490 nm, emission 520 nm). Images were acquired at low magnification (2.5×) and analyzed using Scion Image software. Measurements were performed with the investigator blinded to the treatment group. Tumor area was determined by manually outlining the GFP(+) area and quantification of the surface area (mm^2^) was calculated using computer imaging software. Tumor-associated vessels were defined as those present in the GFP(+) tumor area. These vessels were manually traced by zooming in on the tumor area in the computer-acquired images and the total length of the blood vessels in each tumor area was calculated from the tracings using the computerized imaging software. Vascular length density (VLD) was calculated by dividing the total tumor-associated vessel length by the tumor area (mm/mm^2^).

### Orthotopic tumor growth

Tumor cells (5×10^6^) were inoculated into the mammary fat pad of mice. Three independent single cell clones of each stable cell line were analyzed. Tumor dimensions were measured by caliper twice a week and tumor volume (mm^3^) was estimated using the following formula:
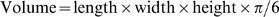
where all linear dimensions are in mm. After the final measurements, the hypoxia marker pimonidazole hydrochloride (NPI, Inc. Belmont, MA) was administered at a dose of 70 mg/kg by i.p. injection one hour prior to sacrifice. The excised tumors were snap frozen in liquid nitrogen.

### Immunocytochemistry

Serial 10 µm sections of frozen mammary fat pad tumor tissues were used. Four randomly selected tumors from each group (pCR3.1 vector and EPOR-R129C) were analyzed in a blinded fashion. Angiogenesis was assessed by microvessel count using anti-CD31 antibody (Dako, Copenhagen, Denmark) as blood vessel marker. Sections were incubated with primary antibody (dilution 1∶50) at room temperature for 60 minutes, and slides were stained by the avidin-biotin method. After the slides were scanned at low magnification (40×), six hot spots (fields with maximum vessel density) per tumor were selected for vessel count. The mean value from six fields was recorded as the microvessel density for each tumor. Staining for the Ki67 proliferation antigen was determined using anti-Ki67 rabbit monoclonal antibody (NCL Ki67, Novocastra, Newcastle, UK) applied overnight at 4°C (dilution 1∶400). Nuclear staining of tumor cells was considered positive for Ki-67. After the slides were scanned at low magnification, the cells with positively stained nuclei were counted in six microscopic fields in each tumor and the Ki67 index was calculated as the ratio of Ki67 positive nuclei per total nuclei. Phospho-ERK1/2 (Thr202/Tyr204) staining was performed using a polyclonal antibody (Cell Signaling Technologies) applied overnight at 4°C (dilution 1∶200). Staining was evaluated at low magnification (20×) by acquiring digital images from 4 or 5 representative phospho-ERK+ fields in each tumor and the extent of staining was quantified for each tumor in a blinded fashion as the percentage of positively stained tumor cells/total tumor cells. Hypoxic tumor areas were determined by immunostaining with monoclonal antibody against pimonidazole adducts (Hypoxyprobe, Chemicon, dilution 1∶50). Digital image analyses were performed under low magnification (4×) to calculate the percentage of pimonidazole positive area /total tumor area by acquiring images of four randomly chosen fields in each tumor.

### Statistical analyses

Data and group measures are reported as mean±s.e.m. The effect of treatments and differences between multiple experimental groups were assessed using repeated measures analysis of variance (ANOVA) and Bonferroni multiple comparisons post-hoc test. The differences between two experimental groups were determined using Student's t-tests. The level of statistical significance was P<0.05.

## Supporting Information

Figure S1 (A) Expression of EPOR-R129C in R3230-GFP cells. Whole cell lysates of R3230-GFP cells transfected with empty vector or EPOR-R129C expression vector were analyzed by Western blotting. Lane 1. Mouse spleen control; Lanes 2–4. Vector-transfected single cell clones; Lanes 5–7. EPOR-R129C-transfected single cell clones. Molecular weight marker is indicated. Arrow indicates ∼66 kDa immunoreactivity consistent with the molecular weight of EPOR. (B) Proliferation of transfected R3230-GFP cells in vitro. Growth curves for EPOR-R129C, R103A-EPO, and empty vector (pcDNA3.1 or pCR3.1) transfected R3230-GFP mammary carcinoma cells were generated by daily cell counts in triplicates (n = 3 experiments in each group). (C) Cell cycle profile of transfected R3230-GFP cells. R3230-GFP cells transfected with EPOR-R129C, R103A-EPO or empty expression vectors were labeled with propidium iodide and the percentage of subdiploid, G1, S, and G2/M populations were determined by flow cytometry. (n = 3 experiments in each group).
(0.90 MB TIF)

Figure S2 Soluble EPOR and neutralizing anti-EPO monoclonal antibody (mab) inhibit EPO-dependent proliferation of hematopoietic cells.- Erythropoietin-dependent 32D cells were cultured in the presence of recombinant EPO and the indicated concentrations of (A) sEPOR or (B) anti-EPO neutralizing mab (mab287). MTT assays were performed and proliferation plotted as a percentage of maximum in the absence of the inhibitor.
(0.60 MB TIF)

Figure S3 (A) Expression of erythropoietin R103A-EPO antagonist in R3230-GFP cells. Cell culture supernatants were analyzed by immunoblotting using anti-EPO polyclonal antibody. Lane 1. Negative control untransfected (UT) R3230-GFP cell supernatant; Lanes 2–4. Empty pcDNA3.1 vector-transfected single cell clones as negative controls; Lanes 5–7.-Single cell clones expressing R103A-EPO in culture supernatants. Molecular weight markers are indicated. Arrow indicates ∼34 kDa immunoreactivity consistent with the molecular weight of EPO and demonstrating the secretion of R103A-EPO into the culture medium. (B) rEPO-induced phosphorylation of ERK1/2 in cells expressing R103A-EPO antagonist. R3230-GFP cells transfected with empty pcDNA3.1 vector or R103A-EPO antagonist were either left untreated as controls (C) or treated with indicated concentration of rEPO for 10 minutes. Whole cell lysates were analyzed by Western blotting using antibodies to detect phospho-ERK1/2 (top panel). The same proteins separated in a duplicate gel demonstrate comparable amount of total ERK1/2 protein (bottom panel).
(0.44 MB TIF)
