# Supplementary figures and images for: Erythropoietin Blockade Inhibits the Induction of Tumor Angiogenesis and Progression
Source: PLoS One. 2007 Jun 20;2(6):e549. doi: 10.1371/journal.pone.0000549 (PMC1891087; doi:10.1371/journal.pone.0000549)

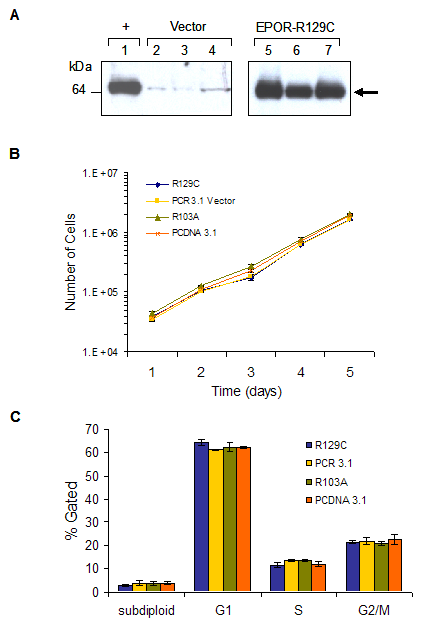

Supplement: Figure S1 — (A) Expression of EPOR-R129C in R3230-GFP cells. Whole cell lysates of R3230-GFP cells transfected with empty vector or EPOR-R129C expression vector were analyzed by Western blotting. Lane 1. Mouse spleen control; Lanes 2–4. Vector-transfected single cell clones; Lanes 5–7. EPOR-R129C-transfected single cell clones. Molecular weight marker is indicated. Arrow indicates ∼66 kDa immunoreactivity consistent with the molecular weight of EPOR. (B) Proliferation of transfected R3230-GFP cells in vitro. Growth curves for EPOR-R129C, R103A-EPO, and empty vector (pcDNA3.1 or pCR3.1) transfected R3230-GFP mammary carcinoma cells were generated by daily cell counts in triplicates (n = 3 experiments in each group). (C) Cell cycle profile of transfected R3230-GFP cells. R3230-GFP cells transfected with EPOR-R129C, R103A-EPO or empty expression vectors were labeled with propidium iodide and the percentage of subdiploid, G1, S, and G2/M populations were determined by flow cytometry. (n = 3 experiments in each group). (0.90 MB TIF) [file pone.0000549.s001.tif]

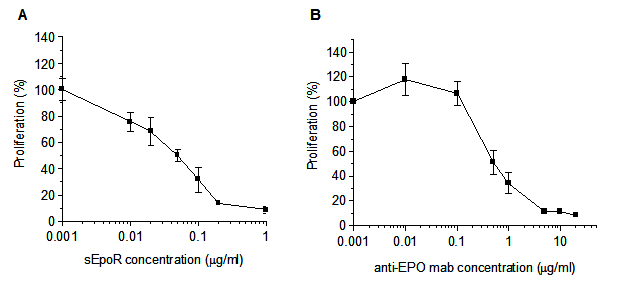

Supplement: Figure S2 — Soluble EPOR and neutralizing anti-EPO monoclonal antibody (mab) inhibit EPO-dependent proliferation of hematopoietic cells.- Erythropoietin-dependent 32D cells were cultured in the presence of recombinant EPO and the indicated concentrations of (A) sEPOR or (B) anti-EPO neutralizing mab (mab287). MTT assays were performed and proliferation plotted as a percentage of maximum in the absence of the inhibitor. (0.60 MB TIF) [file pone.0000549.s002.tif]

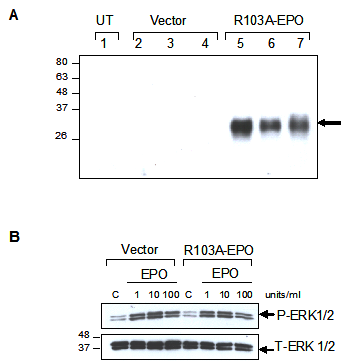

Supplement: Figure S3 — (A) Expression of erythropoietin R103A-EPO antagonist in R3230-GFP cells. Cell culture supernatants were analyzed by immunoblotting using anti-EPO polyclonal antibody. Lane 1. Negative control untransfected (UT) R3230-GFP cell supernatant; Lanes 2–4. Empty pcDNA3.1 vector-transfected single cell clones as negative controls; Lanes 5–7.-Single cell clones expressing R103A-EPO in culture supernatants. Molecular weight markers are indicated. Arrow indicates ∼34 kDa immunoreactivity consistent with the molecular weight of EPO and demonstrating the secretion of R103A-EPO into the culture medium. (B) rEPO-induced phosphorylation of ERK1/2 in cells expressing R103A-EPO antagonist. R3230-GFP cells transfected with empty pcDNA3.1 vector or R103A-EPO antagonist were either left untreated as controls (C) or treated with indicated concentration of rEPO for 10 minutes. Whole cell lysates were analyzed by Western blotting using antibodies to detect phospho-ERK1/2 (top panel). The same proteins separated in a duplicate gel demonstrate comparable amount of total ERK1/2 protein (bottom panel). (0.44 MB TIF) [file pone.0000549.s003.tif]
